# Laughs and Jokes in Assisted Reproductive Technologies: Quantitative and Qualitative Analysis of Video-Recorded Doctor-Couple Visits

**DOI:** 10.3389/fpsyg.2021.648333

**Published:** 2021-04-14

**Authors:** Silvia Poli, Lidia Borghi, Martina De Stasio, Daniela Leone, Elena Vegni

**Affiliations:** ^1^Department of Health Science, University of Milan, Milan, Italy; ^2^Asst Santi Paolo e Carlo, San Paolo University Hospital, Milan, Italy

**Keywords:** assisted reproductive technology, doctor-couple communication, patient centered care, qualitative research, clinical psychology

## Abstract

**Purpose:** To explore the characteristics of the use of laughs and jokes during doctor-couple assisted reproductive technology (ART) visits.

**Methods:** 75 videotaped doctor-couple ART visits were analyzed and transcribed in order to: (1) quantify laugh and jokes, describing the contribution of doctors and couples and identifying the timing of appearance; (2) explore the topic of laughs and jokes with qualitative thematic analysis.

**Results:** On average, each visit contained 17.1 utterances of laughs and jokes. Patients contributed for 64.7% of utterances recorded. Doctor (40.6%) and women (40%) introduced the majority of laughs and jokes. Visits with female physicians had significantly more laughs and jokes than visits with male doctors; no differences were found considering physicians’ age and years of experience, cause of infertility, and prognosis. Laughs and jokes were mainly recorded during history taking and information giving. Four core themes were identified, regarding the topic of laughs and jokes: health status, infertility treatment, organizational aspects, and doctor-patient interaction.

**Conclusion:** Laughs and jokes are common in doctor-couple ART visits and are frequently used during the dialogue, covering a wide range of topics. Results seem to show that laughs and jokes are related to doctor’s personal characteristics (like gender), while are not associated with infertility aspects. Given the complexity of this communicative category, further studies are needed to explore the functions and the effects of laugh and jokes.

## Introduction

The utilization of Assisted Reproductive Technology (ART) is steadily increasing across Europe (Ferraretti et al., 2017) and worldwide (Adamson et al., 2018). ART is a field that poses various challenges at different levels. Infertility, *per se*, causes high levels of stress for most couples (Hasanbeygi et al., 2017). Infertility treatments are a supplementary source of stress for patients because they are long, complex, emotionally and physically demanding and associated with low success rates (Arya and Dibb, 2016; Ferraretti et al., 2017; Domar et al., 2018). As a consequence of distress, patients often discontinue pre-maturely (Gameiro et al., 2012, 2013).

Communication and relational aspects have been considered fundamental to involve patients in the decision-making process and to improve satisfaction and retention in care in ART (Malin et al., 2001; Dancet et al., 2010; Gameiro et al., 2012; Leone et al., 2018). Healthcare workers in ART face various challenges during interaction with the patients: to communicate bad news (e.g., infertility diagnosis, repeated failures in the treatment) (Leone et al., 2017); to address ethical issues (Brezina and Zhao, 2012); to handle patients complaints or distress, which may interfere with treatment routines (Grulke et al., 2009); to manage triadic consultations with two active patients.

Despite these elements highlight the complexity of ART visits, little is known about the communication characteristics of ART visits. In a previous study of our group (Leone et al., 2018), actual communication behavior during doctor-couple interaction was studied using video-recordings. Interestingly, the study highlighted that positive talk (a communication category that includes agreement, approvals, compliments, laughs, and jokes) was the second most representative category for patients (Leone et al., 2018). Generally, positive talk is seen as a response of the patient to the information provided by the physician (Roter, 1997). In a complex and stressful context such as infertility treatment the presence of laughs and jokes, in particular, may seem out of place. However, to date, no study has investigated this communication category in ART yet.

The literature on laughs and jokes in health-care interactions is scarce (Schöpf et al., 2017). Most research has focused on humor which is a complex and dynamic phenomenon that does not have a uniform definition. In the literature different constructs of humor have been investigated such as sense of humor, the personal experience of a humor or humor as a coping style (Schneider et al., 2018).

Regarding the use of humor in clinical interaction, different definitions and identification criteria have been adopted in studies analyzing recorded clinical consultation. For example, laughter has been used as a marker of humor (Sala et al., 2002) and has not been included in the analysis when it was not accompanied to an amusing statement (Schöpf et al., 2017; Phillips et al., 2018). However, laughs and jokes can occur together or be produced independently (Holt, 2011) and both are stereotypically connected with amusement even if they both can have different underlying interactional meanings (Haakana, 2001; Beach and Prickett, 2017; Schöpf et al., 2017). Therefore, the present study aims at investigating laughs and jokes as a broader communicative category, whose incidence in clinical video-recorded visits is still relatively underdetermined, especially in ART visits. Quantification of laughing practices in medical interactions might help to better understand the extent of this phenomenon and its pattern of occurrence, driving attention on its relevance.

The present study aims to investigate more in-depth the use of laughs and jokes during doctor-couple visits in ART. Specifically, the objectives are: (1) to quantify laughs and jokes, describing the contribution of doctors and couples (male and female) and identifying the timing in relation to the phase of the consultation; (2) to assess if there is an association between the number of laughs and jokes and variables like doctor’s age and years of experience, cause of infertility, and prognosis; (3) to explore the thematic topic of laughs and jokes.

## Materials and Methods

The study adopts a quali-quantitative approach.

### Participants and Data Collection

The study is based on the data collected in our previous research (Leone et al., 2018). Participants were recruited from eight Italian ART Centers, through a convenience sample. Patients who agreed to participate filled, before the consultations a sociodemographic form collecting age, level of education and relationship status. Patients’ clinical data regarding the cause of infertility, duration of infertility, and prognosis were collected, after gaining the consent of the patients, from medical records. Physicians also signed an informed consent and completed a sociodemographic form regarding their age and their years of professional experience.

In total, 85 visits were videotaped. For the present study, only the visits with couples (including both male and female patients) were considered, resulting in a sample of 75 consultations (40 first visits and 35 check-ups) for a total of 150 patients and 24 physicians (see Table 1 for socio-demographic characteristics). Visits have been verbatim transcribed.

**Table 1:** Participant sociodemographic and clinical characteristics.

**Patient characteristics**	**Value**
**Gender, n (%)**	
Female	75 (50)
Male	75 (50)
**Participant age, mean years (SD), range**	
Females	36.5 (4.8), 24–49
Males	38.5 (6.8), 24–64
**Participant level of education**, ***n*** **(%)**	
*Females*	
Elementary school	7 (9.3)
High school	38 (50.7)
Graduate and Post-graduate	30 (41)
*Males*	
Elementary school	10 (13.5)
High school	38 (51.4)
Graduate and Post-graduate	26 (35.1)
**Unprotected sex**	
Mean years (SD), range	3.6 (2.9), 0.5–18
**Cause of infertility**, ***n*** **(%)**	
Male	19 (25.7)
Female	19 (27.4)
Mixed	14 (18.9)
Idiopathic	16 (21.6)
Not evaluable	6 (8.1)
**Therapeutic indication**, ***n*** **(%)**	
First level	11 (14.9)
Second level	52 (70.3)
Not recommended	4 (5.4)
Waiting	4 (5.4)
Heterologous	3 (4.1)
**Prognosis**, ***n*** **(%)**	
Favorable	53 (71.6)
Unfavorable	17 (23)
Uncertain	4 (5.4)
**Physician characteristics**	**Value**
**Gender**, ***n*** **(%)**	
Female	15 (62.5)
Male	9 (37.5)
**Participant age, mean years (SD), range**	
Females	46.4 (10.7), 26–62
Males	51.9 (8.4), 41–61
**Participant years in practice, mean years (SD), range**	
Females	16.9 (10.4), 1–33
Males	20.7 (7.8), 11–30

The research project was approved by the Ethical Review Board of the University of Milan and by the Ethical Review Boards of the eight participating ART clinics.

### Procedures

All the utterances coded as “LAUGH” in our previous study (Leone et al., 2018), which used the Roter Interaction Analysis System (RIAS) to analyzed data, have been included. LAUGH in the RIAS coding includes: “trying to amuse or entertain, friendly joke, kidding around, good-natured teasing, morbid jokes and laugh” (Roter and Larson, 2002). However, given the mutually exclusive nature of the RIAS coding system, all visits have been re-analyzed to include jokes and laughter that could have been categorized differently, giving priority to another code (e.g., in the RIAS coding system the utterances of “biomedical information” or “concern” have the priority on the coding of “laughs”). The overall corpus was used for the study.

### Data Analysis

Both quantitative and qualitative analyses were conducted.

As far as quantitative analysis, LAUGH could be a single utterance without the participation of others or could result in a string of back-and-forth comments or laughs between two or more subjects. LAUGH utterances were quantified and compared to the overall utterances of the visits. A ratio between the number of LAUGH utterances of each subject (male patient, female patient, and doctor) and their total contribution to the dialogue was calculated. Utterances were then considered as pieces of conversation, which started with a laughter or a joke and were considered ended after a change of topic or a change of mood of all the three participants (e.g., shift in tone from amused or playful to serious). For each piece of conversation, the researcher recorded who introduced LAUGH (doctor, female patient, or male patient) and how many utterances were produced by the participants in the piece of conversation. The timing was recorded based on the stage of the visit where the exchange took place: introduction, history taking, physical examination, information giving and counseling, closing. Descriptive statistics were calculated for demographic and clinical characteristics and for laughs and jokes utterances. Comparisons between visits with male physicians and visits with female physicians were performed using *t*-test, regarding the number of pieces of conversation and the total number of LAUGH utterances. Pearson correlations were used to analyze relationships between pieces of conversation and LAUGH utterances and continuous variables (physicians’ age and physicians’ years of professional experience). A one-way ANOVA was used to analyze relationships between laughs and jokes variables (pieces of conversation and LAUGH utterances) and variables with more than two levels, namely, cause of infertility and prognosis. All the statistical analyses were performed with SPSS 24 for Windows.

As far as qualitative analysis, each piece of conversation has been transcribed verbatim (Bailey, 2008) and analyzed using inductive thematic analysis (Braun and Clarke, 2006) in order to identify the topic of laughter and jokes. Two authors (S.P and M.DS.) independently read the transcripts and identified an initial list of codes, which were descriptive words or phrases that summarized laughs and joke topics. All the researchers met to compare the emerging code, resolve discrepancies, and categorize the issues into larger codes. The codes were gradually elaborated into themes. In the next stage, themes, sub-themes, and their relations were examined, refined, and checked against the original data set. All researchers discussed until consensus was reached and they were satisfied with the thematic map. Excerpts from the visits were chosen to illustrate each theme.

## Results

### Quantitative Results

Laughs and jokes were present in 72 out of 75 visits; 690 pieces of conversation composed of 1,282 total utterances were recorded. On average, each visit contained 9.2 pieces of conversation (SD = 6.3; range 0–27) and 17.1 utterances (SD = 12.9; range 0–52).

Compared to the total utterances, laughs and jokes account for 2.2% of the dialogue during the visits. Patients contributed for 64.7% of LAUGH (41.9% female, 22.8% male) while doctor accounted for 35.3%. As far as each participant contribution to the dialogue, the percentage of LAUGH utterances compared to the total utterances of the single individual was: 3.9% for the female, 4.4% for the male, 1.2% for the doctor.

Laughs and jokes were mainly initiated by doctors (40.6%) and women (40%); men introduced 19.4%. Half of the LAUGH (53.3%) did not elicit an answer, while the other half was an exchange between the participants composed of two (25.2%), three (12.6%), or more utterances (8.8%).

As far as the timing, frequencies were: 7.8% during introduction, 41.7% history taking, 2.6% physical examination, 46.1% information giving, and counseling, 4.4% closing.

The *t*-tests showed that visits conducted by a female physician had a significantly greater number of total laughs and jokes utterances (*t* = −3.8, *p* < 0.001) and a greater number of pieces of conversations (*t* = −4.5, *p* < 0.001) than visits conducted by a male physician (Table 2).

**Table 2:** Quantitative analysis: association between physicians’ gender and laughs and jokes using *t*-test.

	**Mean (SD)**	***t*-test**	**Mean difference**	**C.I. 95%**	***p*-value**
**Number of laughs and jokes utterances**		−3.8	−9.63	−14.76 to −4.51	<0.001
Females	20.2 (13.6)				
Males	10.5 (8.5)				
**Number of pieces of conversations**		−4.5	−5.46	−7.87 to −3.05	<0.001
Females	11.0 (6.4)				
Males	5.5 (3.9)				

LAUGH utterances did not correlate with physicians’ age (*r* = 0.027, *p* = 0.817) nor with physicians’ years of professional experience (*r* = −0.051, *p* = 0.661). Similarly, the number of pieces of conversation did not correlate with physicians’ age physicians’ (*r* = 0.003, *p* = 0.977) nor with physicians’ years of professional experience (*r* = −0.105, *p* = 0.370).

The number of LAUGH utterances and the number of pieces of conversations did not differ by cause of infertility (respectively *F*= 0.334, *p* = 0.855; *F* = 0.070, *p* = 0.991) and by prognosis (respectively *F* = 0.747, *p* = 0.478; *F* = 0.253, *p* = 0.777).

### Qualitative Results

Four core themes regarding the topic of laughs and jokes during ART visits were identified: health status, infertility treatment, organizational aspects, doctor-patient interaction (Table 3).

**Table 3:** Qualitative analysis: themes, sub-themes, and excerpt from ART visits.

**Main themes**	**Sub-themes**	**Excerpt**	
Health status	Clinical information	F: [I have] bit of gastritis but I think 99.99% of Italians have it (laugh) no I have no health problems	[V19]
	Lifestyle	F: no I weighted 64 (.) 15 years ago M: yes around (.) W: when we met (.) more or less 15 years ago D: Then? What happened? F: Well I went on a diet D: ok ok (gesture with the hand) enough (laugh)	[V54]
	Age	D: you are young because you are my age so (.) you are really young (laugh) F: (laugh) M: (laugh)	[V71]
Infertility	Infertility related information	F: we never used contraception D: ok F: but let us say for a year and a half we- D: you focused F: we focused (laugh) but concentration does not work	[V26]
	Treatment	D: it seems to stab yourself (laugh) F: (laugh) D: if it is inclined it seems:: it still punches a hole but you know (laugh) it is less shocking	[V32]
	Expected outcome and pregnancy	D: it would take the crystal ball to tell you guys go ahead because surely this is the next cycle, or- M: exactly (.) and you do not have the glass ball here (laugh)?	[V35]
Organizational aspects	Set of the visit	D: there are mosquitoes (.) we got company M: eheh	[V38]
	Organization of the ART center	D: you have been here in July F: yes (.) there wa::s D: my colleague (.) F: I thought there was always the same doctor D: basically there is me and two other colleagues Female patient: (laugh) yes so (.) I have to	[V50]
	Legal aspects	D: yes [this drug] is not marketed in Italy (laugh) for this kind of things	[V1]
Doctor-patient interaction	Relational aspects	D: the couple makes me laugh (laugh) an engineer and a psychotherapist F: (laugh) D: the engineer is always precise (.) two plus two while a psychotherapist is mu::ch- M: yes in fact (laugh) let us say two different worlds F: (laugh)	[V21]
	Procedural aspects	D: have you ever been pregnant in your life? F: yes D: when and why? (.) I mean not why (laugh) how it went F: (laugh) M: (laugh)	[V64]
	Chatting	D: did you came by car? F: with the motorcycle Doctor: good because (laugh) knowing the city Female patient: (laugh)	[V2]

*In the table the following transcription conventions and abbreviations have been used: F for female patient; M for male patient; D for Doctor; (.) for silence lasting less than half a second; (.) for silence lasting <1 s; :: for lengthening of a sound; - for cut off or interruption of a sound; [ ] for notes and comments*.

Each main theme is presented with sub-themes and excerpts (with the code of the visit in square bracket) used as examples. For reading the transcript consider the following transcription convention: (.) for silence lasting less than half a second, (.) for silence lasting <1 s, :: for lengthening of a sound; - for cut off or interruption of a sound, [ ] for notes and comments (Bailey, 2008).

#### Theme 1: Health Status

Gaining information from the patients about their health status is a basic goal of the visits. General health of the patients and reproductive health of the couple are one of the topics addressed with laughter and jokes.

*Clinical information*: laughs are often displayed when talking about diseases such as diabetes, previous surgical operations, and exam results.

Female patient: the cholesterol was :: was even higher (laugh) [V43]

*Lifestyle*: Recurring topic are smoke, weight, dietary habits, and physical activity. Either virtuous or negative lifestyle are addressed with laughter.

Doctor: so (.) you smoke 4 or 5 cigarettes a day (.) feeling a lot guilty (laugh) Male patient: (laugh) Female patient: not that much (laugh) [V55]

*Age*: Age is addressed with jokes or laugh. Discussing the role of age in the prognosis, having delayed treatment for a long time, being (or feeling) not young enough for the treatments are recurring topics.

Doctor: okay (.) we are always happy when we see patients born in the 80 s because at least we have-

Female patient: (laugh)

Doctor: (laugh) on our-

Female patient: (laugh) at least we have age [V55]

#### Theme 2: Infertility

*Infertility related information*: Clinical information and exam results regarding infertility diagnosis are jokingly commented or introduced with laughter. During the visits, doctor and patients laugh about the cause of infertility or not knowing the clinical condition underlying infertility; they also laugh about fertility-related clinical conditions (menstruation, number of follicles, retroverted uterus, semen quality) and clinical exam (e.g., spermiogram, hysterosalpingography), commenting on their results or on the procedure (feeling tense or uneasy, fearing or feeling pain).

Male patient: well (.) the problem is me (laugh)

Female patient: (laugh) [V2]

When couple describe their sexual life laughs or jokes also arise, regarding both low and high frequencies of sexual intercourse.

Female patient: when the test signaled that the days of ovulation were over (.) we stopped [having sex] for about a week because-

Doctor: you had enough (laugh) [V67]

Laughs are also displayed when talking about the reproductive history of the couple’s family, such as difficulties during childbearing of their parents or their sibling’s ease of getting pregnant.

Female patient: my sister tried for <3 months and she got pregnant immediately (laugh) [V26]

Laughs and jokes occur also when talking about the couple’s journey to become parents: for how long the couple has been trying to have a baby, unsuccessful assisted reproduction cycles or previous pregnancy.

Female patient: one time too much one time not enough (laugh) from 15 to 1 (laugh) third and last try and then (.) then I do not know what else to do (laugh) [V25]

*Treatment*: laughs and jokes arise during the description of treatment options or during treatment planning.

Female patient: you cannot put a cap on the tuba after you have done the insemination? (laugh) [V50]

Doctors and couples laugh about the number of required exams, or the characteristic of an examination (e.g., spermiogram), or details of interventions (e.g., anesthesia during oocyte retrieval, rest after transfer). Expectation, desire and fear the ART treatment are also introduced and discussed in a light-hearted way.

Male patient: I have made several surgeries and we can say that I am not afraid but (.) aspirating the :: the semen from the testicles yes (laugh) [V36]

The role of the male in the treatment is also addressed with laughter.

Male patient: may I do something?

Doctor: be supportive

Female patient: (laugh) [V56]

*Expected outcome and pregnancy*: many variables play a role in the treatment and, even though pregnancy is a common goal of the couples and the physician, the prognosis is not certain; this is another area of jokes and laughs. To deal with uncertainty, optimism (e.g., how the couples feel) and superstition (e.g., beginning the adoption procedure to increase the chances) are introduced in a playful way. Doctors and couples jokingly comment about the eventuality of having twins, the risk of developing complications, or the couples’ desire about their future child.

W: in case a tumor is formed during pregnancy?

D: you are a little pessimistic

W: yes I am very pessimistic (laugh) [V59]

#### Theme 3: Organizational Aspects

The practical and organizational aspects of a specific ART center and the legislation of treatments are a topic of the visits and a subject of jokes.

*Set of the visit*: The physical elements of the room (temperature, clothes hangers, lights) and their utilization during the visits are commented with laughs. Doctors and couples laugh about the slowness of the computer, or the obligation to insert the data in the informatic system of the center.

Doctor: I need to register you in a :: medical record that is electronical but unfortunately on one hand I am illiterate on this matter and on the other hand the desk is small I have to turn back and forth (laugh)

Female patient: (laugh)

Male patient: (laugh) [V19]

*Organization of the ART center*: The delivery and the continuity of care and the characteristic of the ART center is commented (e.g., being visited by different doctors). The cost of treatments is also a topic of laughs.

Doctor: and then the cost

Male patient: yes the cost (.) that was the question indeed

Doctor: that is the bad news usually it is communicated by the secretary (laugh)

Female patient: (laugh)

Male patient: (laughing) because first they tell you everything and then they tell you the cost or else you do not listen to them

Doctor: no no (laugh) [V8]

*Legal aspects*: Doctors and patients laugh about the obligation to sign the informed consent, and the imposition of treatment restrictions (egg donation, embryo freezing); differences between foreign Countries are also addressed.

Male patients: there has been a new law (laugh) was it the day before yesterday uh? [V71]

#### Theme 4: Doctor-Patient Interaction

This theme includes laughs and jokes concerning the actors of the visits or their interaction during the encounter.

*Relational aspects*: The relation between doctors and patient is commented with jokes. Doctor and couples laugh about their past interaction or their personal characteristics, preferences and inclination also commenting on the way this impact on the others and on the treatments.

Doctor: what could we do to deal with him [referring to the male patient]?

Female patient: it is impossible to deal with him (laugh) [V8]

*Procedural aspects*: In this case, the relation is not the focus but is the frame in which laughs are displayed. Situations and events that happen during the visits are commented and laughed.

Doctor: lay down

Female patient: now I will start coughing (laugh) [V21]

*Chatting*: Laughs arise also when talking about topics unrelated to the visit such as the weather or the private life of patients and doctors (holidays, hobbies, hometown).

Male patient: I work as a computer consultant

Doctor: One of those that when you call them you do not understand anything of what they tell you

Male patient: (laugh) [V6]

## Discussion

The present study aimed to quantitatively and qualitatively explore the use of laughs and jokes during doctor-couple visits in the ART field.

The quantitative findings showed that laughs and jokes were registered in the vast majority (96%) of the visits and were largely used during the interaction, with an average rate of 17 utterances per encounter. This finding is only partially consistent with the literature related to other settings as 94% of diabetes visits contained amusing comment (Schöpf et al., 2017), while only 60% of primary care and specialty care visits contained reciprocated and shared amusement (Phillips et al., 2018). These studies reported lower frequencies of target events per visit, ranging from two instances (Phillips et al., 2018) to six (Sala et al., 2002; Schöpf et al., 2017). One explanation could be the different definition and inclusion criteria, however, it could be hypothesized that the high frequency of laughs and jokes is due to the different setting analyzed: ART treatments are long and complex and infertility is a stressful and burdensome issue, therefore laughs and jokes might be used to strengthen the relationship between participants (Martin et al., 2003), create a partnership and to produce a more relaxed atmosphere (Joshua et al., 2005). Accordingly, the present study highlighted that laughs and jokes occurred in all moment of the dialogue from the introduction to the closing of the visit and that about half of the laughs and jokes registered were reciprocated resulting in an exchange between participants. It is interesting to note that half of the laughs occur in the interaction between at least two of the presents while the others appear as singular interventions. This may suggest than laughing is not always a way to communicate: people may not decode the messages in the same way: amusing comment could be unacknowledged or misinterpreted by the listener and laughs could be displayed by one person after interpreting as funny something that was not intended to be; laughs could also be an expression of stress or embarrassment (i.e., nervous laugh) experimented by one of the parties (Gervais and Wilson, 2005).

As observed in other studies (Sala et al., 2002; Schöpf et al., 2017), our results highlight that laughs and jokes are more frequently introduced and used by patients than physicians; however, one study did not found differences (Phillips et al., 2018). Nevertheless, considering each individual separately, doctors and female patients equally produce and initiate laughs and jokes, while male patients laugh and joke less frequently. As reported by Leone et al. (2018), male patients are less active than doctors and female patients. According to the limited scientific literature on men in ART, men would like equal involvement between partners and a more balanced dialogue with professionals (Mikkelsen et al., 2013) but they subjectively feel dismissed and disconnected from the treatment and unacknowledged in the dialogue (Mikkelsen et al., 2013; Arya and Dibb, 2016; Leone et al., 2018). Despite the wish to be more involved, medical professionals communicate primarily with female patients (Mikkelsen et al., 2013; Leone et al., 2018) probably because biologically women play a bigger part in the treatments (Gdanska et al., 2017). In the present study, comparing laugh utterances with the total talk of the individual, our results highlight that male patients seem to use laughs and jokes quite consistently in their discourse. In other words, male patients are less talkative, but their interventions are more frequently made of laughs or jokes. Laughter and jokes are a way to enhance relationship-building (Sala et al., 2002) and it could be hypothesized that male patients use it as a way to intervene in the conversation and feel more involved.

Visits with female physicians had significantly more laughs and jokes than visits with male doctors. This finding is consistent with the results found by Sala et al. (2002). The authors suggested that physicians play a role in setting the tone of the conversation, and, in the case of female physicians, patients are more encouraged to use laughs and jokes. This could be further explained by the literature regarding humor style that highlights that woman usually engage in positive forms of humor such as affiliative and self-enhancing humor (Martin et al., 2003). It would be interesting to investigate gender differences in terms of humor style during clinical encounters.

Interestingly, our results highlight that the presence of laughs and jokes is not associated with the cause of infertility or the prognosis, indicating that patients and physicians laugh regardless of the expected outcome of infertility treatments.

Qualitative analysis highlights that a wide range of topics are addressed with laughter encompassing clinical, personal, and contextual topics. Every aspect of ART care might be jokingly commented, even serious and sensitive topics such as unsuccessful assisted reproduction cycles, which can be surprising. Nevertheless, humor has been found in a variety of hospital settings including palliative care in relation to death and dying (Adamle and Ludwick, 2005; Dean and Gregory, 2005); it could be a way to discuss difficult topics in a less threatening way. This way of dealing with emotional issues could be the reason why funny comments arise also in relation to sexual behavior, which is a sensitive topic that can create embarrassment. Besides talking about serious topics, doctors and couple joke on topics that are not strictly related to treatments such as the context in which the visits is being held (e.g., the temperature of the room), the event that happens during the visits (e.g., receiving a call) or personal and general topics (e.g., hobbies). This could be a way to relieve the tension, taking a break from the seriousness of the visit and to foster relationship-building promoting connectedness and warmth (Phillips et al., 2018). Interestingly, the relationship between doctors and couples is also a topic addressed with laughter and jokes; this underlines the importance of the relation in ART treatment. In fact, a good relationship with the doctor is one of the major reported needs of couples in ART (Malin et al., 2001; Hasanbeygi et al., 2017; Borghi et al., 2019). Joking on personal characteristic and making funny comments about peculiar dynamics between the participant may minimize status differences and create a sense of partnership.

Even if laughs and jokes may be constructive, their positive effect should not be taken for granted. For example, the overuse of laughs and jokes as a strategy to deal with emotion-provoking topics could have a paradoxical effect: instead of making the discussion easier, it could divert the dialogue leading to the avoidance of the issue (Joshua et al., 2005). Physicians should, therefore, pay attention if an issue is addressed multiple times with a facade of amusement as it could hide deep concerns that need further investigation (Bennett, 2003). Likewise, being more conscious of the effect of laughs and jokes could be fundamental in a triadic communication, where jokes may have different effects on the participants.

The present study is preliminary and presents some limitations. First, the study is observational and based on a previously collected dataset. Second, although our data derived from video-recorded visits, non-verbal clues were not always available due to the position of the camera, reducing the contextual elements that are needed to interpret the underlying dimensions of laughs and jokes. Moreover, the present study was not designed to explore the function of laughs and jokes that are, however, a crucial aspect in order to understand the multifaceted interactional role of laugh within the ART visits and eventually connect it to the humor literature. In this sense, the perspective of doctors and patients should be taken into account in future studies, in order to confirm the intent of laughs and jokes. Another limit to the present study is that data on psychological characteristics of the couple or of the physicians were not included in the study design; moreover, clinical outcomes such as retention in care or adherence have not been investigated.

Finally, visits have been collected in a specific context and, as laughs and jokes are often influenced by culture (Granek-Catarivas, 2005) it would be useful to repeat the study in other countries.

However, to the best of our knowledge, this is the first study to explore the use of laughs and jokes in assisted reproductive technology visits and one of the few studies addressing laughs and jokes in doctor-patient interaction using video-recordings of naturally occurring communication (Schöpf et al., 2017).

The present study highlighted that laughs and jokes are frequently used during doctor-couple ART visits addressing a wide range of topic and therefore this complex communication category should be further explored. Future studies are needed to clarify the functions of laughs and jokes in doctor-patients communication and to understand their effect on patients’ clinical (e.g., satisfaction, retention in care or adherence) and psychological (e.g., depression, anxiety) variables.

## Data Availability Statement

The data analyzed in this study is subject to the following licenses/restrictions: Data are available on request due to privacy or other restrictions. Requests to access these datasets should be directed to Silvia Poli, silvia.poli@unimi.it.

## Ethics Statement

The studies involving human participants were reviewed and approved by Ethical Review Board of the University of Milan and by the Ethical Review Boards of the eight participating ART clinics. The patients/participants provided their written informed consent to participate in this study.

## Author Contributions

SP and MD contributed to the transcription of visits. SP, MD, and LB contributed to the data analysis. SP and LB contributed to the draft of the work. All authors contributed to the conception and design of the work, interpretation of data, revised draft of the work critically and gave their final approval of the version to be published.

## Conflict of Interest

The authors declare that the research was conducted in the absence of any commercial or financial relationships that could be construed as a potential conflict of interest.
